# Displaced supracondylar femoral fractures: Clinical and radiographic outcomes in children aged 4–10 years treated with Kirschner wires and hip spica cast

**DOI:** 10.3389/fped.2023.1086831

**Published:** 2023-01-24

**Authors:** Yanhui Jing, Bo Ning, Yueqiang Mo, Dahui Wang

**Affiliations:** Department of Orthopaedics Surgery, National Children’s Medical Center & Children’s Hospital of Fudan University, Shanghai, China

**Keywords:** pediatric, displaced, supracondylar femoral fractures, Kirschner wire, hip spica cast

## Abstract

**Background:**

Supracondylar femoral fractures (SFFs) are uncommon in children but can cause several abnormalities. Although several methods have been employed to treat these fractures, no accepted standard has been established.

**Objectives:**

To investigate the clinical and radiographic outcomes of displaced SFFs treated with Kirschner wires (K-wires) and hip spica casts in children aged 4–10 years.

**Methods:**

We retrospectively reviewed 22 displaced SFFs (mean age, 6.7 years; range, 4–10 years) in patients who underwent surgical treatment with K-wires and hip spica casts. The patients were followed-up frequently, radiographically and clinically between January 2014 and February 2019. Postoperative healing and functional results were elevated according to the radiographic and clinical measures.

**Results:**

Fifteen boys and seven girls were included in this study. All patients except two (91%), underwent closed reduction and stabilization of the fractures. The mean follow-up duration was four years (range, 2–5 years). All fractures showed clinical and radiological evidence of union 4–8 weeks after surgery. At the most recent check-up, all patients reported being pain-free and had returned to normal activities. The mean Knee Society Score was 95.41 at the final follow-up. According to the radiologic criteria, 18 of the 22 patients (81.8%) obtained excellent results, 3 (13.6%) had good results, 1 (4.5%) had a fair result, and none had poor results.

**Conclusion:**

Satisfactory clinical and radiological results can be expected in children aged 4–10 years using a combination of K-wires and hip spica cast fixation.

## Introduction

Supracondylar femoral fractures (SFFs) are rare in children. They are also associated with complications, such as limb length discrepancy and deformity of the femur. In an investigation of 115 patients, Smith et al. (1) discovered that supracondylar fractures accounted for 12% of all femoral fractures and that only approximately 50% of these fractures were displaced. Moreover, an increasing tendency towards surgical treatment of pediatric fractures has been reported (2).

Many methods have been employed in managing displaced supracondylar fractures, including external or plate fixation, percutaneous crossing wiring, and others (1–5, 9). However, there is no universally accepted conclusion regarding the optimal treatment. Numerous variables influence the decision to fixate, including the age and size of the child, associated injuries, location and pattern of the fracture, and social situation of the child (3–5). Kirschner wires (K-wires), as a less invasive technique, have been reported for the treatment of displaced SFFs in younger patients (3, 6), but whether this treatment can be safely used in older patients remains uncertain. This study's primary goal was to retrospectively assess the clinical and radiographic outcomes of displaced SFFs treated with K-wires and hip spica cast.

## Materials and methods

**Figure 1:**
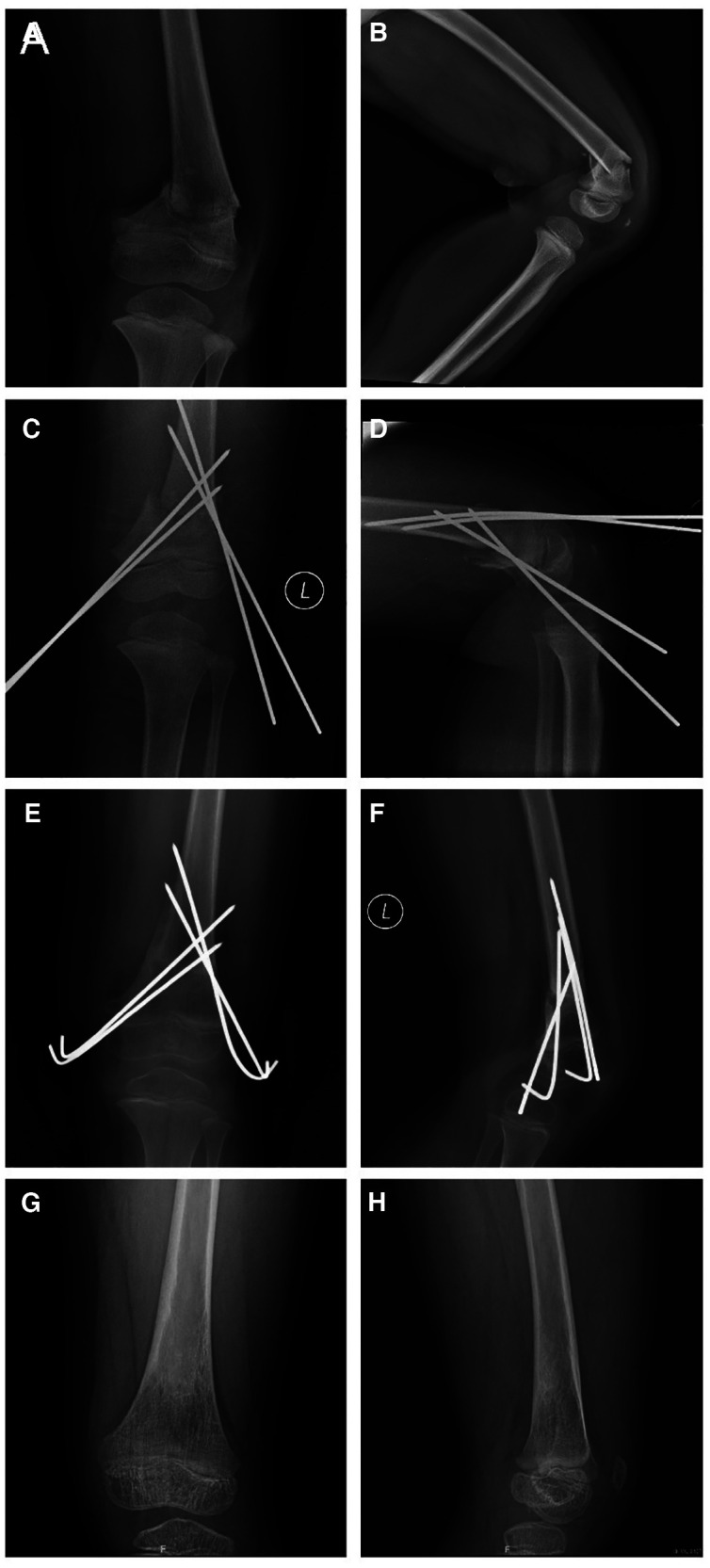
Outcome in a 4-year-old girl with a displaced supracondylar femoral fracture. Anteroposterior (**A**) and lateral radiographs (**B**) obtained before surgery. Intraoperative anteroposterior (**C**) and lateral radiographs (**D**). Anteroposterior (**E**) and lateral radiographs (**F**) obtained 1 month after surgery, and showing the fracture has healed. Anteroposterior (**G**) and lateral radiographs (**H**) obtained 1 year after surgery, which showed limb alignment was normal and epiphyseal development was normal.

The medical records were reviewed for the following information: sex, age, height, weight, fractured limb, mechanism of injury, associated injury, and fracture type. All records of patients treated at the authors' institution from January 2014 to February 2019 were reviewed. To be included in the study, patients were required to meet the following criteria: (1) SFF as determined by radiographs, (2) surgical treatment with K-wire and hip spica cast fixation, and (3) follow-up for at least 2 years. A supracondylar fracture was defined as a fracture spanning a distance equal to the condylar width from the joint line (Figure 1). The exclusion criteria were as follows: (1) pathological or open fracture, (2) intra-articular distal femoral fracture, and (3) incomplete medical data. This retrospective study was approved by the ethics review board of the authors' institution.

**Figure 2:**
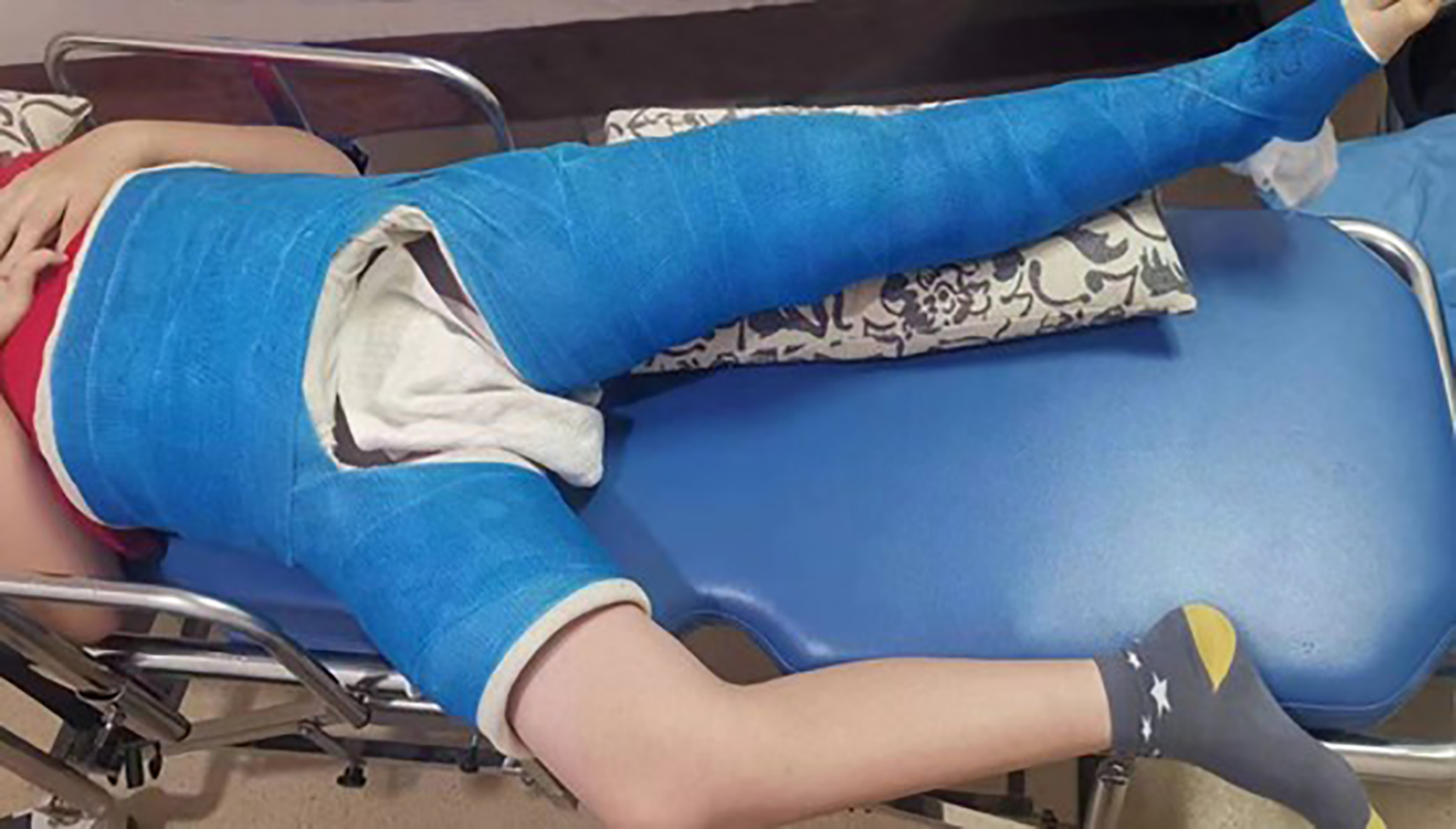
A 9-year-old boy, treated with Kirschner wire internal fixation followed by a one-and-one-half spica plaster cast.

All surgeries were performed under general anesthesia under the guidance of a C-arm machine. After general anesthesia and muscle relaxation, the patient was placed in the supine position. First, a closed reduction was attempted. Reduction was performed using manual traction during flexion in anteriorly displaced fractures and extension in posteriorly displaced fractures. Following the application of longitudinal traction on the leg to keep the fragments in proper alignment, three or four Crossed K-wires with a diameter of 1.6 mm or 2.0 mm were then used in percutaneous cross-pinning *via* the epicondyles under the supervision of an image intensifier. When closed measures of reduction were ineffective (two cases, 9%), open reduction was performed.A longitudinal incision about 10 cm was made laterally at the distal part of the femur, then, the iliotibial band and lateral femoris muscle were separated to expose the fracture site. The fracture was reduced under direct vision.Following the procedure, the pins were left with percutaneous protrusion. All patients received a one-and-one-half hip spica cast (hip and knee extension positions) for 4–8 weeks after surgery (Figure 2), after which the K–wires were taken out and functional rehabilitation started. The children were shown how to perform exercises and were referred to a physical therapist if function was not improved within a week. All patients resumed walking 8–10 weeks after surgery.

All patients were followed up every two weeks with x-rays until the fracture healed, and then monthly for the next 3 months until functional recovery was satisfactory. Afterwards, the patients were followed-up every 6 months until complete clinical and radiographic union was attained, or for at least two years. At each follow-up appointment, the injured knee's range of motion (ROM) was measured and contrasted with that of the unaffected side. Knee function was assessed using the new Knee Society Score (KSS) at the last follow-up (7).

## Result

This study assessed 22 children aged 4–10 years with displaced SFFs who met the inclusion criteria and were treated with K-wires (Table 1). There were 15 boys (68.2%) and 7 girls (31.8%) with a mean age at trauma of 6.7 years (range, 4–10 years) and a mean weight of 25.4 kg (range,18−42 kg). The right femur was injured in 14 patients (63.6%), and the left femur in 8 patients (36.4%). The mechanism of injury included motor vehicle accidents in six patients (27.3%), falls from bicycles in four (18.2%), sports in nine (40.9%), and accidental falls in three (13.6%). There were 13 transverse-type fractures (59%) and 9 oblique-type fractures (41%). According to the AO classification (8), 15 patients (68.2%) had type 33A1 fractures, six (27.3%) had 33A2, and only one (4.5%) had 33A3.

**Table 1:** Characteristics of patients with supracondylar femoral fractures treated by K-wires.

No.	Sex	age	Side	Weight (kg)	Type of fracture	Operation type	K-wire size/number	KSS	Score
1	M	4.5	R	22	Transverse	Close	1.6/4	94	Excellent
2	M	7	R	20	Oblique	Close	1.6/4	96	Good
3	F	5	R	18.5	Transverse	Close	1.6/3	95	Excellent
4	M	9.2	R	35	Oblique	Close	2.0/4	95	Excellent
5	M	4.5	L	18.5	Transverse	Close	1.6/4	97	Excellent
6	F	7	L	18	Oblique	Close	1.6/4	96	Excellent
7	M	6.5	R	24.5	Transverse	Close	1.6/4	95	Excellent
8	M	7	R	32	Transverse	Close	1.6/4	95	Excellent
9	F	6	R	25	Transverse	Close	1.6/4	97	Excellent
10	M	8.7	R	35	Oblique	Close	2.0/4	98	Excellent
11	M	6.5	L	23	Transverse	Close	2.0/4	96	Excellent
12	M	7	R	26	Oblique	Open	1.6/4	95	Excellent
13	F	5.5	L	30	Oblique	Close	1.6/3	95	Good
14	M	7	R	22	Transverse	Close	2.0/4	95	Excellent
15	M	7.5	R	28	Transverse	Close	2.0/4	94	Excellent
16	M	9.5	R	42	Oblique	Open	2.0/4	96	Good
17	F	9	L	26	Transverse	Close	2.0/4	95	Fair
18	M	8	L	27	Oblique	Close	2.0/4	96	Excellent
19	F	7	R	25	Transverse	Close	1.6/3	94	Excellent
20	F	6	L	20	Transverse	Close	1.6/4	96	Excellent
21	M	4	L	21	Oblique	Close	1.6/3	93	Excellent
22	M	5	R	20	Transverse	Close	1.6/3	96	Excellent

KSS, knee society score.

### Clinical outcome

All patients initially underwent closed reduction; in two cases (9.1%), open reduction was performed after closed reduction was unable to achieve the desired results. The mean surgical time was 45 min (range, 30–90 min). All patients had their implants removed after radiological evaluation 4–8 weeks postoperatively. ROM limitation was not observed in any of the children at the most recent follow-up visit, and the mean peak KSS was 95.41.

### Radiographic outcomes

All patients were obtained physical examination about the difference in lower extremity, from the greater trochanter of the femur to the tip of the lateral malleolus.At the last follow-up, no patient showed a difference of >1 cm from the contralateral limb. Leg length disparity and sagittal and coronal deformities were the radiological criteria used for radiological evaluation (9). The results according to the radiological criteria are shown in Table 2. Eighteen fractures had no radiological deformities and were assessed to be excellent. Two fractures with a 4-degree varus deformity and one with a 10-degree flexion deformity, were assessed as good. One patient had a 7-degree varus deformity, and was assessed as having fair results. There were no poor results recorded.

**Table 2:** Grading of radiologic results.

	Leg length discrepancy	Sagittal deformity	Coronal deformity	Fractures (*n* = 22)
Excellent	<1 cm	—	—	18 (81.8%)
Good	<1 cm	<11	<6	3 (13.6%)
Fair	1 cm–2 cm	11–20	6–10	1 (4.5%)
Poor	>2 cm	>20	>10	0

### Complication

Three patients had superficial infection in the needle tract, which was controlled by dressing changes and oral antibiotics. No nerve or vascular injuries were observed postoperatively. There was no delayed union or non-union. One patient had a residual varus deformity, one patient had a residual valgus deformity, and one patient had a mild flexion deformity.

## Discussion

Displaced SFFs are far less common than diaphyseal fractures and account for less than 18% of all pediatric femoral fractures (1). Various methods have been used to treat displaced SFFs; however, there is no technique frequently suggested in the literature. The patient's age and physique, the injury site, the fracture pattern, and whether there are any additional injuries should all be considered while deciding on the best course of action (2–4). For younger patients, the recommended course of action is to regulate alignment, length, and rotation; reduce joint stiffness; and ensure good functional recovery with minimal complications. Treatment should be accommodating for the patient and their family in the interim and should have the least detrimental psychological effects (3–5).

To sustain reduction, Bor et al. (10) presented a unique approach combining temporary intraoperative exterior fixation and permanent internal fixation. External fixation was performed after the fractures were securely fixed with a plate. This method is particularly suitable for the treatment of pathological fractures in older children. Some scholars have advocated for the use of locking plates or external fixation (11–13). The locking plates must be opened to expose the fractured part. Surgical trauma is more aggressive and affects blood flow at the fracture site. The damage to the epiphyseal plate and epiphysis was greater. There is an increased risk of postoperative epiphyseal abnormalities, delayed healing of fractures, nonhealing, or infection after surgery. External fixation has been widely used in femoral fractures with minimal invasion; however, the risks of pin-tract infection, refracture, bending, and delayed union have been reported in the literature, and the hardware needs to be removed for the second operation.

Parikh et al. (14) reported that elastic intramedullary nails are an important option for treating supracondylar fractures in children, and can achieve ideal results. However, elastic intramedullary nails are prone to needle tail irritation, poor anti-rotation ability, and a high incidence of complications such as nail removal and nail site irritation. The fracture site shown on the radiograph was more proximal and was not a true supracondylar fracture of the femur.

As a minimally invasive method of internal fixation, K-wires are widely used in the treatment of pediatric fractures. Kirschner wire and hip cast fixation techniques are relatively simple, have a shorter learning curve, and are friendlier to the chief resident and attending physician. Therefore, it is the first choice of treatment for most surgeons in our institute. To the best of our knowledge, this is the largest case series focusing on displaced SFFs treated with K-wires, and emphasizing the clinical and radiological characteristics and management methods. Close or open reduction and percutaneous K-wire fixation have been reported for displaced SFFs in pediatric patients in some studies, but mainly in very young children (15, 16). Li et al. (16) reported that 12 children with displaced supracondylar fractures underwent closed reduction with K-wire fixation. Satisfactory results were obtained during follow-up. However, the ages of the selected patients in their study, were significantly different from that of the patients in this study. Owing to concerns about sufficient mechanical support and anti-rotational stability, which indicate the risks of delayed union or malunion, patients under 4 years of age were selected in his study. However, the patients in our study were 4–10 years of age, with significant differences in age and weight. During surgery, we were also concerned about the stability of the K-wire; therefore, we used a one-and-one-half spica cast instead of a brace or single-leg cast for each patient. These methods achieved good stability and no nonunion was observed in our study. The rate of excellent and good radiographic results was 95.5%. In case number 17, a 9-year-old girl, an infection was identified near the needle tract at the 2-week postoperative follow-up, so we partially released the cast to examine the wound, and needle-tail infection characterized by red and swollen exudation was detected. The infection was treated with continuous dressing changes and oral antibiotics. In the subsequent follow-up, due to loosening of the cast, the girl presented with mild secondary displacement of the fracture, and there was a residual 7° varus deformity.

A total of 9 patients with 10 displaced supracondylar fractures were reported by Butcher et al. (9), who also noted two cases of 10° flexion deformity and one case of an 11° to 20° sagittal deformity with a 6° to 10° valgus deformity. This additional information may have been caused by the complexity of the injuries and availability of external fixation. Considering the relative instability of the K-wire and the poor compliance of children, a one-and-one-half spica plaster was chosen instead of an above-knee plaster cast in our patients. A spica plaster provides better stability and complements the K-wire. In the group of patients managed in our institution, there was only one case of re-displacement due to cast loosening during follow-up. Active functional exercises were initiated once the cast had been removed. According to our follow-up data, the long-term functional rehabilitation was satisfactory. None of the patients experienced hyperextension or pain while walking after regaining full range of motion at the knee joint.

According to Smith et al. (1), closed reduction and percutaneous fixation with crossed wires needed to cross either the growth plate or the articular cavity to achieve fixation, where there were risks of damaging the femoral vessels medially and purulent arthritis for the former option, and for the latter, inflammatory arthritis. Epiphyseal damage is unlikely to occur as a result of K-wire penetration. In an experimental investigation on rabbits (17) the growth plate needed to be destroyed in 7% of its cross-sectional area to permanently disrupt growth and shorten limbs. The diameter of the smooth K-wires employed in our investigation ranged from 1.6 to 2.0 mm, which is less than 1% of the cross-sectional diameter of the epiphysis. The findings of this study are consistent with those of earlier studies on pediatric SFFs with percutaneous pinning, in that neither growth disruption nor significant limb length disparity was observed (18).

Firstly, a small sample size decreased the power of our statistical analysis. However, displaced supracondylar femoral fracture in pediatric practice is rare, and the results of the present study provide a new insight for such diseases. A multicenter prospective study may be a better way of studying these rare but challenging injuries. Secondly, lack of a control group of other treatment options and brief follow-up time also affected the statistical efficiency. Longer-term close follow-ups until skeletal maturity of those patients with good outcomes are necessary in our subsequent works. Finally, the intrinsic limits of a retrospective study in a single center cannot be avoided completely. However, this study shows that a percutaneous crossed K-wire combined with a hip spica cast is an effective treatment for misplaced SFFs in children aged 4–10 years.

## Conclusion

We reported a single-stage protocol for treatment of displaced supracondylar femoral fractures in children aged 4–10 years. Satisfactory radiological and clinical outcomes can be expected by percutaneous crossed K-wire and hip spica cast fixation.

## Data Availability

The original contributions presented in the study are included in the article/Supplementary Material, further inquiries can be directed to the corresponding author.

## References

[1] Smith NC Parker D McNicol D. Supracondylar fractures of the femur in children. J Pediatr Orthop. (2001) 21:600–3. 11521026

[2] Kosuge D Barry M. Changing trends in the management of; children’s fractures. Bone Joint J. (2015) 97-B:442–8. 10.1302/0301-620X.97B4.34723 25820880

[3] Pretell MJ Rodriguez MJ Andres EE. Surgical approaches for open reduction and pinning in severely displaced supracondylar humerus fractures in children: a systematic review. J Child Orthop. (2010) 4:143–52. 10.1007/s11832-010-0242-1 21455471 PMC2839861

[4] Ehlinger M Ducrot G Adam P Bonnomet F. Distal femur fractures. Surgical techniques and a review of the literature. Orthop Traumatol Surg Res. (2013) 99:353–60. 10.1016/j.otsr.2012.10.014 23518071

[5] Periasamy K. Supracondylar fracture of the femur in children. WebmedCentral TRAUMA. (2010) 1(9):WMC00594. 10.9754/journal.wmc.2010.00594

[6] Li J Yue C Wang H-Q, Guo X-K, Chen K-L, Ma J-W, et al. External fixation and Kirschner wires in the treatment of paediatric displaced supracondylar femur fractures. J Child Orthop. (2020) 14:293–8. 10.1302/1863-2548.14.200050 PMC7453171 32874362

[7] Scuderi GR, Bourne RB, Noble PC, Benjamin JB, Lonner JH, Scott WN. The new knee society scoring system. Clin Orthop Relat Res. (2012) 470:3–19. 10.1007/s11999-011-2135-0 22045067 PMC3237971

[8] Slongo TF Audige L. Fracture and dislocation classification compendium for children: the AO pediatric comprehensive classification of long bone fractures (PCCF). J Orthop Trauma. (2007) 21(10 suppl):S135–60. 10.1097/00005131-200711101-00020 18277238

[9] Butcher CC Hoffman EB. Supracondylar fractures of the femur in children closed reduction and percutaneous pinning of displaced fractures. J Pediatr Orthop. (2005) 25(2):145–8. 10.1097/01.bpo.0000149860.50400.92 15718890

[10] Bor N Rozen N Dujovny E, Rubin G. Fixator-Assited plating in pediatric supracondylar femur fractures. Glob Pediatr Health. (2019) 6:1–5. 10.1177/2333794X19843922 PMC6484234 31041364

[11] Li J Guo X Wang HQ. Locking plate versus external fixation in the treatment of displaced femoral supracondylar fracture in children. J Child Orthop. (2020) 14:293–8. 10.1302/1863-2548.14.200050 32576269 PMC7310551

[12] Bible JE Mir HR. External fixation: principles and applications. J Am Acad Orthop Surg. (2015) 23:683–90. 10.5435/JAAOS-D-14-00281 26306568

[13] Eidelman M Keren Y Norman D. Correction of distal femoral valgus deformities in adolescents and young adults using minimally invasive fixator-assisted locking plating (FALP). J Pediatr Orthop B. (2012) 21:558–62. 10.1097/BPB.0b013e328358f884 22960367

[14] Parikh SN Nathan ST Priola MJ, Eismann EA. Elastic nailing for pediatric subtrochanteric and supracondylar femur fractures. Clin Orthop Relat Res. (2014) 472(9):2735–44. 10.1007/s11999-013-3240-z 23955195 PMC4117889

[15] Su Y Nan G. ORIF With percutaneous cross pinning via the posterior approach for paediatric widely displaced supracondylar femoral fractures. Injury. (2016) 47(6):1242–7. 10.1016/j.injury.2016.02.024 26997133

[16] Li J Ma J Guo X Yue C Chen K Wang J Closed reduction with crossed Kirschner wire fixation for displaced supracondylar femoral fractures in young children. Medicine (Baltimore). (2020 Mar) 99(13):e19666. 10.1097/MD 32221095 PMC7220454

[17] Dahl WJ Silva S Vanderhave KL. Distal femoral physeal fixation: are smooth pins really safe? J Pediatr Orthop. (2014) 34:134–8. 10.1097/BPO.0000000000000083 23965910

[18] Sink EL Faro F Polousky J Flynn K Gralla J. Decreased complications of pediatric femur fractures with a change in management. J Pediatr Orthop. (2010) 30:633–7. 10.1097/BPO.0b013e3181efb89d 20864844

